# Supplementary material for: Patterns of Intron Gain and Loss in Fungi
Source: PLoS Biol. 2004 Nov 30;2(12):e422. doi: 10.1371/journal.pbio.0020422 (PMC532390; doi:10.1371/journal.pbio.0020422)
Supplement: Table S1 — Also available at http://genes.mit.edu/NielsenEtAl/. (4.3 MB ZIP). [file pbio.0020422.st001.zip › NielsenEtAl/html/410.html]

AN6066.1.NCU00166.1.MG05741.1.FG07259.1


```
 CLUSTAL W (1.82) Multiple Sequence Alignments - Introns Inserted


Sequence 1: NCU00166.1	228 aa
Sequence 2: FG07259.1	202 aa
Sequence 3: MG05741.1	265 aa
Sequence 4: AN6066.1	207 aa
Alignment Length: 283 aa
Number Identitical Residues: 73 aa
Alignment Score (without introns) 3546


MG05741.1 	MS0PPTPLSLRSFNQPMLRNGAALMSFAARHSTRASSAPPVHLGAAEMATTAAQAAGSRR
NCU00166.1	--~--------------------------------------------MTDSTPKQR---A
FG07259.1 	--~--------------------------------------------MSSSAQNEL---R
AN6066.1  	--~--------------------------------------------MAAISRACR----
          	                                               *:  :        

MG05741.1 	LE1NPVLF2VCDMQDKFRNAIWEFDKI2VLTSQKVIRAAHLLSIPIYATTQNRAKLGPTV
NCU00166.1	FE1KPALF2VCDIQEKFRNAIYEFDKV2VSTTTKLLRFAHALHIPIYVTTQTRAKLGETV
FG07259.1 	FK1NPRIF2VCDIQEKFRKVIYEFDSI2VLTTQKLVKFANSLSVPVITTTQTSAKLGPTV
AN6066.1  	IR1NPAVF2ICDMQEKFEKAIYEFPKI2VTTTTKLLRAANTLSIPIFVTTQNRARLGATV
          	:. :* :* :**:*:**.:.*:** .: * *: *::: *: * :*: .***. *:** **

MG05741.1 	PELEELLTAAG-------------PLVREHSDKTKFSMWTPSVQRTLAARDLLGGSGSGR
NCU00166.1	SELQPFLTQKDGGGSSSSSSSSLHPLVAADLDKTLFGMYTPSLASLFSSSPASI-----P
FG07259.1 	SSLAQLLP-------------------SAPHDKTKFSMVIPSVVADLPPN----------
AN6066.1  	PVLQQLLNGPN---------------VRADIDKTLFSMVTPEIDGLLPVPKKGE----AL
          	. *  :*   .                    *** *.*  *.:   :.          . 

MG05741.1 	VTVALVGIESHICVTQTALDLLR--AGHGVYVLADGVSSCNKEEVPLALARLRAE-GAVV
NCU00166.1	SHIALVGIESHICVTQTCLALRKKHPNTKIYVIADGVSSCNKEEVPVALARLRQE-GVTV
FG07259.1 	SEIALVGIESHICITQTALDLRN--AGHKVYVIADGVSSCNPREVGIALDRLRAEPGITV
AN6066.1  	MDAIIVGIETHICVTQTTLDLLER--GHRVYVLVDGVSSINAEERGIALARLRDA-GAIV
          	    :****:***:*** * * .   .  :**:.***** * .*  :** ***   *  *

MG05741.1 	TSSEAWLYEVMGDAEIPEFRSIVGLVKKTSADTAAVLQALRPARM--
NCU00166.1	TTSEGWMYECMGDAGIPEFREVVKIVKETAEGTREALRALGPLGAKI
FG07259.1 	TSSESWMYECVGDASHSAFKGLFGVVKESMADTKKVWQTLPPE-SKI
AN6066.1  	TTSESILFEILGDANHQHFKAIAGLVKETKEETKEALEKLSKI----
          	*:**. ::* :***    *: :  :**::   *  . . *
```
